# A Novel Molecularly Imprinted Electrochemiluminescence Sensor Based on Mxene Quantum Dots for Selective Detection of Oseltamivir in Biological Samples

**DOI:** 10.3390/molecules30010152

**Published:** 2025-01-02

**Authors:** Wei Guo, Shiqiang Yan, Chaoqiang Xiao, Dayong Shi, Qing Hua, Xiaowen Hao, Wenjuan Zhang, Xuming Zhuang

**Affiliations:** 1 Shandong Dyne Marine Biopharmaceutical Co., Ltd., Weihai 264300, China; ysqiang@fudan.edu.cn (S.Y.); xiaochaoqiang@dynemed.com (C.X.); sdyg21@163.com (D.S.); 2 School of Chemistry and Chemical Engineering, Yantai University, Yantai 264005, China; 13844630958@163.com (Q.H.); 20240023@ytvc.edu.cn (X.H.); yeah19981122@163.com (W.Z.)

**Keywords:** oseltamivir, Mxene, molecular imprinting technology, electrochemiluminescence

## Abstract

Oseltamivir is a drug that has been widely used to prevent and treat influenza A and B. In this work, an ultrasensitive, simple, and novel electrochemiluminescence (ECL) sensor combined with molecularly imprinted polymers (MIP-ECL) based on a graphene-like two-dimensional material, Mxene quantum dots (MQDs) was constructed to selectively detect oseltamivir. A molecularly imprinted polymer membrane containing an oseltamivir template was constructed by electropolymerization and elution of modified MQDs on a glassy carbon electrode. Under optimized experimental conditions, the MIP-ECL sensor could detect oseltamivir in the range of 10^−10^ to 10^−6^ M (R^2^ = 0.9816), with a low limit of detection of 6.5 × 10^−11^ M (S/N = 3), and the recovery rates of oseltamivir in biological samples were 92.21–104.2%, with relative standard deviations of 3.70%~5.70%. The developed MIP-ECL sensor provides a new idea for detecting oseltamivir, which was successfully applied to the determination of oseltamivir in serum samples, indicating great potential for application in clinical diagnostics.

## 1. Introduction

Oseltamivir is a neuraminidase inhibitor that treats or prevents influenza A and B viruses, was first synthesized in 1996 [1], and oseltamivir was originally developed on the basis of zanamivir, which is poorly absorbed by living organisms [2,3]. On the basis of zanamivir [4], oseltamivir was designed according to the molecular structure of the natural neuraminidase substrate and the spatial structure of the neuraminidase catalytic center. Studies have shown that oseltamivir can prevent influenza by inhibiting the escape of mature influenza viruses from host cells [5]. Some investigations on the possible side effects of oseltamivir have revealed that not only the toxic side effects of the drug itself but also the metabolites of the drug will have an increasingly serious impact on environmental pollution, and its biological activity has potential dangers to the survival and development of aquatic organisms [6,7,8]. Therefore, monitoring and analysis of oseltamivir is highly important. At present, the commonly used methods for oseltamivir detection include electrochemical methods [9], ultraviolet-visible spectrophotometry [10], and chromatography-mass spectrometry [11]. Although the above techniques have high sensitivity and selectivity, the sample pretreatment cost is high, and the steps are complex, requiring a long test time. Therefore, establishing a fast, simple, accurate, sensitive, and economical analytical method for oseltamivir analysis is highly important.

Compared with other commonly used analytical methods, electrochemical biosensors are widely used in modern analytical chemistry because of their technical simplicity, high portability, sensitivity, cost-effectiveness, and practical feasibility [12]. Electrochemiluminescence (ECL) technology, as a combination of electrochemical and chemiluminescence processes, has common advantages, such as high sensitivity, a wide kinetic range, simple operation, and good stability [13]. Compared with chemiluminescence [14], ECL has better spatiotemporal controllability for light emission. Compared with photoluminescence analysis [15], ECL does not need to excite the light source, effectively avoids the interference of stray light, and increases the sensitivity of the analysis. ECL sensors have been widely used in the analysis field. Molecularly imprinted polymers (MIPs) are polymers processed via molecular imprinting techniques that leave cavities in the polymer matrix and have an affinity for selected “template” molecules [16]. Molecular recognition sites for specific target molecules are created in MIPs to obtain materials with high selectivity for specific target molecules. In recent years, MIP sensors have attracted attention because of their ultrahigh selectivity and have been widely used in isolation, disease diagnosis, drug delivery, single-cell analysis, bioimaging, cancer diagnosis, and therapy [17,18,19]. ECL sensors combined with MIPs (MIP-ECL) not only have excellent selectivity but also have the advantages of high sensitivity and simple preparation, which has become a hot trend in the research and application of MIPs.

In recent years, graphene-like two-dimensional materials have been widely used in optoelectronics, catalysts, biosensors, and supercapacitors because of their high specific surface area, excellent thermal conductivity, and other properties. A new class of graphene-like two-dimensional materials (Mxene) composed of transition metal carbides, nitrides, or carbon nitrides has attracted extensive attention. Jae-Min et al. provide a comprehensive review of the latest advances in Mxene-based sensor technology, provide broad insights into previous and existing technologies, and highlight current deficiencies in Mxene sensors and future directions [20]. Li et al. constructed a novel ECL biosensor based on glutathione-coated Mxene quantum dots (MQDs) for the detection of miRNA221 [21]. Li et al. developed a novel ECL biosensor based on MQDs and SnS_2_ nanosheets/lipid bilayers to detect the gastric cancer marker miRNA-27a-3p at concentrations ranging from 1 fM to 10 nM [22].

In this work, we developed a novel ultrasensitive MIP-ECL sensor based on MQDs for oseltamivir detection. MQDs synthesized with reference to previous work have high and stable ECL signals. MIP films were prepared via the use of oseltamivir as a template molecule and *o*-phenylenediamine (o-PD) as a functional monomer during electropolymerization. After electropolymerization, the oseltamivir, o-PD, and MQDs modified glass carbon electrode (GCE) is eluted to form a MIP film containing an imprinted cavity, which can specifically recognize the template molecule (Oseltamivir). The sensor has high sensitivity and selectivity for oseltamivir detection. Therefore, the proposed MIP-ECL sensor with a strong and stable cathode ECL signal can achieve trace detection of oseltamivir in biological serum.

## 2. Results and Discussion

### 2.1. Characteristics of MQDs

**Figure 1:**
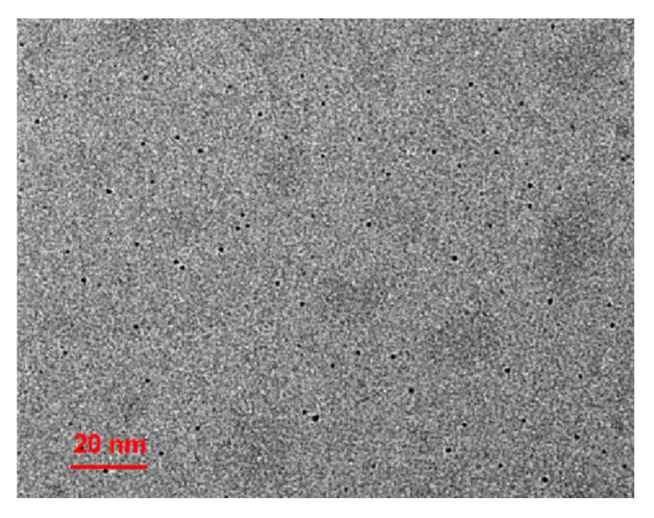
TEM images of the MQDs.

**Figure 2:**
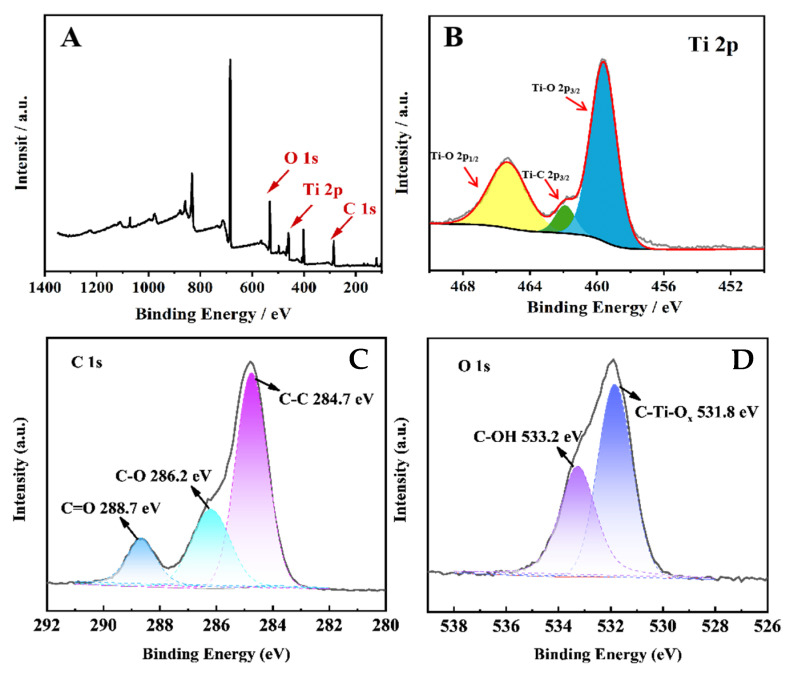
(**A**) XPS survey spectrum and (**B**) Ti 2p, (**C**) C 1s, and (**D**) O 1s spectra of the MQDs.

Figure 1 shows TEM images of the MQDs. As shown in Figure 1, the distribution of the quantum dots is uniform and circular, and the particle size is approximately 3 nm. The elements in the MQDs were analyzed via XPS. As shown in Figure 2A, the peaks at 532.1 eV, 458.9 eV, and 284.9 eV belong to O 1s, Ti 2p, and C 1s in the quantum dots, respectively. Figure 2B shows the XPS spectral fitting of the Ti 2p peak. Two characteristic peaks are found at binding energies of 458.3 eV, 462.5 eV, and 465.8 eV, corresponding to Ti 2p_3/2_ and Ti 2p_1/2_ spin-orbital split photoelectrons, respectively [23]. As shown in Figure 2C, the peaks at 284.7, 286.2, and 288.7 eV correspond to the C-C/C-H, C-O and C=O of the MQDs, respectively. The O 1s XPS spectrum (Figure 2D) has two peaks: those of C-Ti-O_x_ and C-OH are 531.8 eV and 533.2 eV, respectively.

### 2.2. Characterizations of Different Modified Electrodes

**Figure 3:**
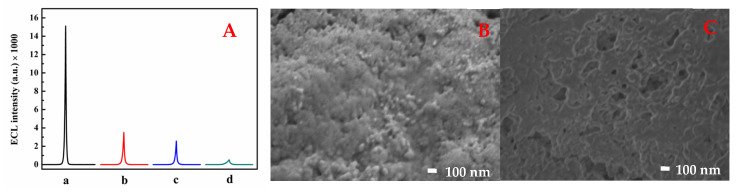
(**A**) ECL intensity of the (a) MQDs/GCE, (d) MIP-ECL/GCE, (b) elution and (c) recombination of oseltamivir in the MIP-ECL/GCE. SEM images of the MIP-ECL/GCE after polymerization (**B**) and after elution (**C**).

As shown in Figure 3A, the MQDs/GCE (curve a) has extremely high ECL strength. In contrast, the MIP-ECL/GCE has a weak ECL strength (curve d), which may be due to the nonconducting substance that covers the MIP-ECL/GCE, leading to electron transfer between the MIP-ECL/GCE and K_2_S_2_O_8_ being blocked. However, after the extraction of oseltamivir from the MIP-ECL/GCE (curve b), the intensity of the ECL peak slowly increased compared with that of curve d, since the formation of many cavities caused redox reactions between the MQDs and K_2_S_2_O_8_ to resume. Moreover, the ECL intensity decreased after absorption by oseltamivir (curve c), which was attributed to oseltamivir being embedded in the cavity. As shown in Figure 3B, after the electropolymerization of the MQDs/GCE, the MIP membrane was tightly bound to the surface of the electrode, resulting in a rough surface with tightly bound particles of different sizes. As shown in Figure 3C, oseltamivir was washed off after eluting the polymerized imprinted film, and a lamellar cavity structure formed on the surface of the MQDs/GCE. According to the SEM results, a molecularly imprinted film was successfully constructed on the surface of the electrode, which guaranteed the subsequent construction of the MIP-ECL sensor.

### 2.3. Electropolymerization Procedure

**Figure 4:**
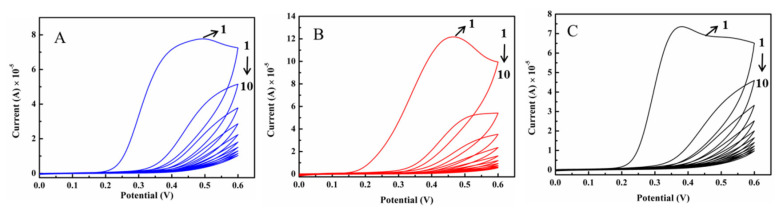
The electropolymerization of o-PD with oseltamivir at (**A**) the bare GCE (**B**) MQD/GCE and (**C**) without oseltamivir at the MQD/GCE by using CV (vs. Ag/AgCl (saturated)).

Figure 4 shows the electropolymerization process by using CV at various modified GCEs. As depicted in Figure 4A, o-PD and oseltamivir were electropolymerized onto the bare GCE. The first CV curve exhibited an oxidation peak at 0.5 V, and as the number of scans increased from 1 to 10, the peak of oxidation gradually decreased. The o-PD was coated onto the bare GCE to form polymeric substances. As shown in Figure 4B, compared with that of the bare GCE, the CV curve of o-PD was electropolymerized onto the MQDs/GCE without oseltamivir, and an oxidation peak at 0.46 V was observed because the MQDs facilitated electron transfer. The o-PD and oseltamivir were electropolymerized onto the MQDs/GCE to form OOM/GCE, as shown in Figure 4C. Compared with the above MQDs/GCE without oseltamivir, the CV had an oxidation coefficient of 0.37, which was attributed to the overlap of the peaks of o-PD and oseltamivir. As the number of scanning cycles increases, the number of peaks decreases gradually, possibly because o-PD and oseltamivir form insulating materials on the MQDs/GCE.

### 2.4. Optimization of Experimental Conditions

**Figure 5:**
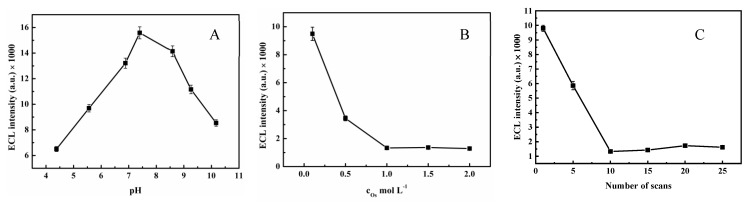
Optimization of the experimental conditions: (**A**) pH; (**B**) oseltamivir concentration; (**C**) number of electropolymerization cycles.

The optimized experimental conditions were a prerequisite for obtaining a good sensor. Figure 5A shows that various pH values affect the performance of the MIP-ECL/GCE, and when the pH was 7.4, the ECL signal was high. In addition, the effects of the concentration of oseltamivir and the number of electropolymerization cycles strongly influence the experimental results. Therefore, we investigated the effect of the oseltamivir concentration during the electropolymerization process (Figure 5B). As the concentration increased (0.1, 0.5, 1.0, 1.5, and 2.0 M), the ECL intensity decreased, so a concentration of 0.1 mol L^−1^ was selected as the optimal concentration for the influence experiments. As shown in Figure 5C, the ECL intensity at 10 electropolymerization cycles was stable; thus, we chose 10 as the optimal number of electropolymerization cycles.

**Figure 6:**
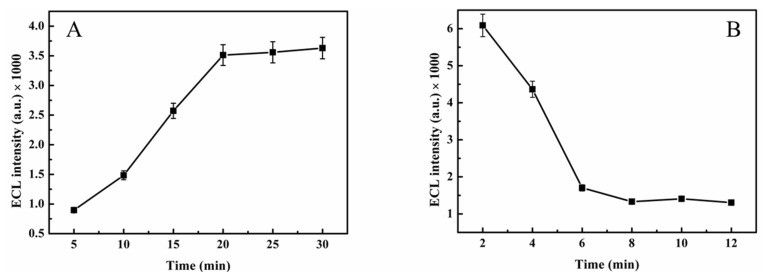
The effects of (**A**) elution time and (**B**) absorption time on changes of the ECL intensity.

The number of electropolymerizations is also an important factor in the study of sensors. Different electropolymerization times can result in polymers with different morphologies. As shown in Figure 6A, the ECL values increased with increasing elution time until 20 min, at which point the ECL intensity was stable. Thus, elution at 20 min was the optimal elution time. For comparison, absorption times of 2, 4, 6, 8, 10, and 12 min were selected for comparison with 2 min. We selected 6 min as the experimental condition because the ECL intensity was stable.

### 2.5. Possible ECL Mechanism

Next, the mechanism of the MIP-ECL sensor is explored. When the ECL test is performed using an initial negative potential (−2 V), charge injection reduces the MQDs on the GCE surface to negatively charged free radicals MQDs^•−^. At the same time, S_2_O_8_^2−^ is reduced on the electrode to produce the strong oxidizing agent SO_4_^•−^, and then MQDs^•−^ is oxidized by SO_4_^•−^ to form excited-state MQDs*. The excited MQDs* then return to the ground state in an unstable way to release photons and produce light. The above mechanism is shown in the following equation [24]:

MQDs + e → MQDs^•−^, (1)

S_2_O_8_^2−^ + e → SO_4_^2−^ +SO_4_^•−^, (2)

MQDs^•−^ + SO_4_^•−^ → MQDs* + SO_4_^2−^, (3)

MQDs* → MQDs + *hv*, (4)

### 2.6. ECL Analytical Performance of the Constructed Sensor for Oseltamivir Detection

**Figure 7:**
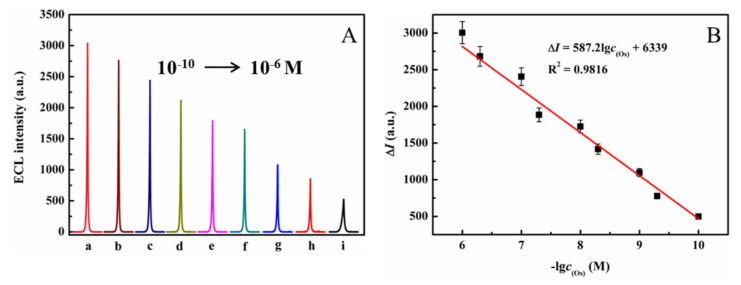
(**A**) ECL curves of the sensor with different concentrations of oseltamivir (a to i: 1 × 10^−10^; 5 × 10^−9^; 1 × 10^−9^; 5 × 10^−8^; 1 × 10^−8^; 5 × 10^−7^; 1 × 10^−7^; 5 × 10^−6^; 1 × 10^−6^ M); (**B**) the corresponding linear relationship for detecting oseltamivir.

To identify the sensitivity of the constructed ECL sensor, quantitative measurements were obtained by determining the optimal concentrations of oseltamivir. The ECL intensity decreased as the oseltamivir concentration increased from 10^−10^ to 10^−6^ M (Figure 7A), and the fitted linear data of the ECL intensity and the oseltamivir concentration could be expressed as ∆*I* = 587.2lg*c*_(Oseltamivir)_ + 6339, R^2^ = 0.9816 (Figure 7B). The limit of detection (LOD) was calculated to be 6.5 × 10^−11^ M (S/N = 3).

**Figure 8:**
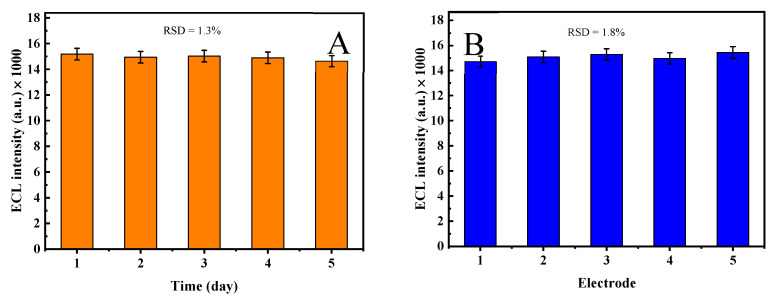
(**A**) Stability and (**B**) reproducibility of the MQD/GCE.

Moreover, to further check the stability of the MQD/GCE (Figure 8A), we chose one MQD/GCE for ECL testing over five consecutive days, and the relative standard deviation (RSD) was 1.3%, indicating that the MQD/GCE has high stability. For reproducibility evaluation (Figure 8B), we used five MQD/GCEs and recorded them at room temperature, and the RSD was calculated to be 1.8%, which indicated that the MQD/GCE has good reproducibility.

### 2.7. Stability and Selectivity of Oseltamivir Detection

**Figure 9:**
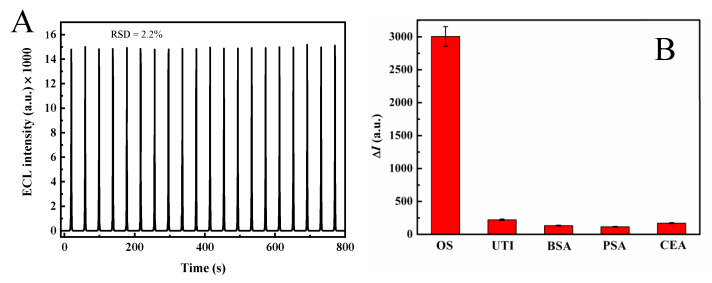
(**A**) ECL stability of the MQDs/GCE. (**B**) Specificity of the ECL sensor for oseltamivir (10^−10^ M), and interfering substances, including UTI, BSA, PSA, and CEA (10^−10^ M).

Stability and specificity have been investigated as two crucial properties of sensors. As shown in Figure 9A, the relatively stable ECL response was monitored for 20 cycles of ECL scans with RSD = 2.2%, revealing the excellent stability of the MQDs/GCE. As shown in Figure 9B, we selected various interfering substances, such as ulinastatin (UTI), BSA, prostate-specific antigen (PSA), and carcinoembryonic antigen (CEA), and the ECL signal only responded to oseltamivir. These results demonstrate the superior specificity of the proposed sensor.

### 2.8. Application for Oseltamivir Detection in Actual Samples

To evaluate the potential of the ECL sensor for oseltamivir detection, standard addition recovery experiments were performed. The experimental results are shown in Table 1. The recoveries varied from 92.21% to 104.2% and RSDs (3.70%~5.70%), which suggested that these values were within the acceptable range and proved that the ECL sensor has prominent potential in actual sample analysis.

## 3. Materials and Methods

### 3.1. Materials

LiF and Ti_3_AlC_2_ were obtained from Shanghai Aladdin Biochemical Technology Co. (Shanghai, China). K_2_S_2_O_8_ and o-PD were obtained from Sinopharm Chemical Reagent Co., Ltd. (Tianjin, China). Bovine serum albumin (BSA) and fetal bovine serum (FBS) were obtained from Aladdin Reagent Co., Ltd. (Shanghai, China). A phosphate buffer solution (PBS, 0.1 M, pH 7.4) was prepared from a standard mixture of 0.1 M Na_2_HPO_4_ and 0.1 M KH_2_PO_4_. All the chemicals were of analytical grade and were used without further purification, and the solutions involved in these experiments were prepared with ultrapure water (18.25 MΩ cm).

### 3.2. Apparatus

Scanning electron microscopy (SEM) images and transmission electron microscopy (TEM) images were obtained on JSM-7900F and JEM-2100 (200 kV) instruments, respectively (JEOL Ltd., Tokyo, Japan). X-ray photoelectron spectroscopy (XPS) measurements were performed on a Thermo ESCALAB-250 instrument (Thermo Fisher Scientific, Waltham, MA, USA). The fluorescence emission spectra were obtained on an F-4700 fluorescence spectrophotometer (HITACHI, Tokyo, Japan). The cyclic voltammetry (CV) and ECL signals were characterized via an MPI-E ECL detection system (Xi’an Remax Electronic Science and Technology Co. Ltd., Xi’an, China). A Nicolet 5700 Fourier transform infrared (FT-IR) spectrometer (Thermo Electron Corporation, Waltham, MA, USA) was used to obtain FT-IR spectra. The ultraviolet-visible (UV–vis) absorption spectrum was examined with a UV-2450 UV–vis spectrophotometer (Shimadzu, Kyoto, Japan).

### 3.3. Preparation of MQDs

Briefly, 1 g of LiF was slowly added to 20 mL of HCl (30% *v*/*v*) and stirred for 5 min. Then, Ti_3_AlC_2_ was added to the above mixture and stirred for 24 h at 35 °C. After being centrifuged with ultrapure water at 3500 rpm, the mixture was maintained at pH > 6. Finally, after ultrasonication under an N_2_ atmosphere for 1 h, the liquid supernatant was obtained after it was centrifuged for 1 h at 3500 rpm, and the resulting sample was denoted as Mxene nanosheets.

First, 20 mL of Mxene nanosheets was mixed with ammonia water, and the pH was adjusted to 9. The prepared solution was subsequently added to the reactor, which was heated at 100 °C for 6 h. The mixture was subsequently washed and centrifuged three times with distilled water for 30 min at 10,000 rpm. Finally, the pale-yellow supernatants were MQDs (0.5 mg mL^−1^) [25].

### 3.4. Preparation of the MIP-ECL/GCE

Five microlitres of MQDs were coated on the clean surface of the GCE (diameter of 3 mm, Chenhua Instruments, Shanghai, China) and then dried at room temperature to obtain the MQDs/GCE.

A buffer solution of HAc-NaAc containing O-PD (5 mM) and oseltamivir (5 mM) was electropolymerizated to obtain the MIP-ECL/GCE [18], which included oseltamivir. The potential ranged from 0–0.60 V, the scan speed was 50 mV s^−1^, and the sweep was turned 10. Then, under magnetic stirring conditions, the prepared MIP-ECL/GCE was washed with CH_3_OH/H_2_O (*v:v* = 2:1) for 15 min to remove the Oseltamivir template. Afterward, we washed with water and dried the sample to obtain the MIP-ECL/GCE. Under the same conditions, the NIP-ECL/GCE (NIP: nonimprinted polymer) was prepared without adding oseltamivir.

### 3.5. ECL Measurements

The MIP-ECL/GCE ECL sensing platform served as the working electrode, a platinum wire served as the counter electrode, and an Ag/AgCl electrode served as the reference electrode. The MIP-ECL/GCE was immersed in a series of concentrations of Oseltamivir with HAc-NaAc buffer solution, washed with water, and dried at room temperature. Five milliliters of K_2_S_2_O_8_ (0.05 M, pH = 7.4) as the coreagent was prepared from 0.1 M phosphate buffer solution (PBS), which was provided by a voltage range from −2.1 to 0 V at a scanning rate of 100 mV s^−1^ and the voltage of the photomultiplier tube (PMT) was 800 V.

## 4. Conclusions

In summary, we proposed a novel MPI-ECL sensor for the detection of oseltamivir based on a molecularly imprinted polymer on a GCE. First, we prepared the molecular imprinting template of the MIP-ECL/GCE with good ECL intensity, and as the concentration of oseltamivir further increased, the ECL intensity decreased. Therefore, a sensitive “signal-off” sensor for quantitative determination of oseltamivir has been constructed with a wide detection range from 10^−10^ M to 10^−6^ M with an LOD of 6.5 × 10^−11^ M. Additionally, the molecular imprinting template was successfully used to detect oseltamivir in actual serum samples, indicating a novel and highly selective method for detecting oseltamivir.

## Figures and Tables

**Table 1:** Actual sample analysis of oseltamivir in FBS by the enhanced chemiluminescence (ECL) sensor.

Samples	Add (nM)	Founded (nM)	Recovery/%	RSD/%
FBS	10	9.22 ± 0.41	92.21	5.70
	1	1.04 ± 0.05	104.2	4.12
	0.1	0.102 ± 0.004	101.9	3.70

## Data Availability

Data are contained within the article.
